# GRP78 Knockdown Enhances Apoptosis via the Down-Regulation of Oxidative Stress and Akt Pathway after Epirubicin Treatment in Colon Cancer DLD-1 Cells

**DOI:** 10.1371/journal.pone.0035123

**Published:** 2012-04-18

**Authors:** Yu-Jia Chang, Yi-Ping Huang, Zih-Ling Li, Ching-Hsein Chen

**Affiliations:** 1 Graduate Institute of Clinical Medicine College of Medicine, Taipei Medical University, Taipei, Taiwan; 2 Department of Surgery, College of Medicine, Taipei Medical University, Taipei, Taiwan; 3 Department of Surgery, Taipei Medical University Hospital, Taipei, Taiwan; 4 Cancer Research Center, Taipei Medical University Hospital, Taipei, Taiwan; 5 Department of Microbiology, Immunology and Biopharmaceuticals, College of Life Sciences, National Chiayi University, Chiayi City, Taiwan; Duke University Medical Center, United States of America

## Abstract

**Introduction:**

The 78-kDa glucose-regulated protein (GRP78) is induced in the cancer microenvironment and can be considered as a novel predictor of responsiveness to chemotherapy in many cancers. In this study, we found that intracellular reactive oxygen species (ROS) and nuclear factor erythroid 2-related factor 2 (Nrf2) nuclear translocation were higher in GRP78 knockdown DLD-1 colon cancer cells compared with scrambled control cells.

**Methodology/Principal Findings:**

Treatment with epirubicin in GRP78 knockdown DLD-1 cells enhanced apoptosis and was associated with decreased production of intracellular ROS. In addition, apoptosis was increased by the antioxidants propyl gallate (PG) and dithiothreitol (DTT) in epirubicin-treated scrambled control cells. Epirubicin-treated GRP78 knockdown cells resulted in more inactivated Akt pathway members, such as phosphorylated Akt and GSK-3β, as well as downstream targets of β-catenin expression. Knockdown of Nrf2 with small interfering RNA (siRNA) increased apoptosis in epirubicin-treated GRP78 knockdown cells, which suggested that Nrf2 may be a primary defense mechanism in GRP78 knockdown cells. We also demonstrated that epirubicin-treated GRP78 knockdown cells could decrease survival pathway signaling through the redox activation of protein phosphatase 2A (PP2A), which is a serine/threonine phosphatase that negatively regulates the Akt pathway.

**Conclusions:**

Our results indicate that epirubicin decreased the intracellular ROS in GRP78 knockdown cells, which decreased survival signaling through both the Akt pathway and the activation of PP2A. Together, these mechanisms contributed to the enhanced level of epirubicin-induced apoptosis that was observed in the GRP78 knockdown cells.

## Introduction

GRP78 is a chief regulator of endoplasmic reticulum (ER) function. The roles of GRP78 include (1) protein folding and assembly, (2) targeting misfolded protein for degradation, and (3) ER Ca^2+^-binding and control of the activation of transmembrane ER stress sensors. Moreover, due to its anti-apoptotic property, GRP78 is induced in a wide variety of cancer cells and drug-resistant cancer cells [1]. Interestingly, GRP78 expression is significantly stronger in colon cancer than in colon adenoma and normal tissue [2]. In addition, a recent study showed that GRP78 knockdown not only efficiently suppressed the proliferation of RKO colon cancer cells but also induced the early apoptosis of the cells [3]. Moreover, GRP78 downregulation has been shown to result in colon cancer sensitization to paclitaxel-induced apoptosis [4]. Taken together, these reports highlight the important role of GRP78 in therapeutic treatment.

Several anticancer agents result in oxidative stress by producing reactive oxygen species (ROS) and inducing cytotoxicity and apoptosis in cancer cells [5]. Oxidative stress that occurs during chemotherapy, however, may interfere with the cytotoxic effects of anticancer agents, which depend on the rapid proliferation of cancer cells for optimal activity [5]. Other studies have also illustrated that moderate oxidative stress can stimulate the proliferation and survival of cancer cells through conditioning mechanisms, whereas the enhancement of ROS overproduction by prooxidants under severe oxidative stress can result in apoptosis and cell death [6]. In redox signaling, Nrf2 plays a critical role in the transcription of a series of genes that contribute to phase II/III enzymes and the defense against oxidative stress [7]. There is increasing evidence for frequent mutations of Nrf2 in human cancers, which result in a large amount of Nrf2 nuclear translocation and lead to the constitutive expression of cytoprotective and detoxification genes. The growth advantages and resistance to apoptosis provided by these genes provide chemoresistance during therapy [8]. Other reports have also illustrated that treatment with chemotherapeutic drugs activates the Nrf2 pathway, which induces cytoprotective genes and modulates chemosensitivity in colon cancer cells [9]. Therefore, the inhibition of Nrf2 nuclear translocation can be presumed to suppress cell proliferation and enhance apoptosis in cancers. Taken together, these studies show that oxidative stress and redox regulation play important roles in chemotherapy.

Akt is an apoptotic regulator that is activated in many cancers and may promote drug resistance *in vitro* [10]. Survival signals induced by various receptors are primarily mediated by Akt; thus, the Akt pathway may promote resistant phenotypes [11]. The recurrent deregulation of the Akt survival signaling pathway in cancers has resulted in significant interest among researchers attempting to block this pathway for treatment purposes [12]. Hence, inhibition of the Akt pathway is being considered as a novel strategy to sensitize cancer cells to anticancer drugs [13].

Epirubicin is a doxorubicin derivative anthracycline analog that has a better therapeutic index than doxorubicin [14]. With the same dose, epirubicin elicits lower hematologic and cardiac toxicity than doxorubicin [15]. The mechanism of epirubicin on cytotoxicity appears to involve the production of ROS [16]. To date, the detailed anticancer mechanism of epirubicin in GRP78 knockdown colon cancer cells has not been elucidated. In the present study, we were interested in understanding the anticancer effect of epirubicin on the apoptotic potential of GRP78 knockdown in human colon DLD-1 cancer cells. We were also interested in understanding the apoptotic mechanism of GRP78 knockdown on oxidative stress, redox regulation and the Akt survival pathway during epirubicin treatment so that a guide may be developed for additional antineoplastic therapeutics for human colon cancers.

## Materials and Methods

### Cell line, reagents and chemicals

The human colon cancer cell line DLD-1 was obtained from the Bioresource Collection and Research Center (Hsinchu, Taiwan). RPMI-1640 medium and fetal bovine serum (FBS) were obtained from Hyclone (South Logan, UT) and Gibco Inc. (Freehold, NJ), respectively. Arrest-In transfection agent was obtained from Open Biosystems Inc. (Huntsville, AL). The Nrf2 siRNA, scrambled siRNA, primary antibodies against p-Akt (Thr308), Akt, Nrf-2, GSK-3β, β-catenin, SP-1 and GAPDH were obtained from Santa Cruz Biotechnology, Inc. (Santa Cruz, CA). The Akt kinase assay kit was acquired from Cell Signaling Technology (Boston, MA). The Bio-Rad protein assay reagent was purchased from Bio-Rad Laboratories (Richmond, CA). The Ser/Thr phosphatase assay kit was purchased from Millipore Corporation (Billerica, MA). Propyl gallate (PG), dithiothreitol (DTT), propidium iodide (PI), 2′,7′-dichlorodihydrofluorescein diacetate (DCFH-DA), DNase-free RNase A, dimethyl sulfoxide (DMSO), trypan blue, anti-actin primary antibody and other chemicals were purchased from Sigma Chemical Co. (St. Louis, MO).

### Cell culture and treatment

DLD-1 cells were cultured in RPMI-1640 medium containing 10% FBS. The stock solution of epirubicin was dissolved in DMSO, and the treated concentration (500 ng/ml) was prepared in RPMI-1640 medium.

### Generation of GRP78 knockdown DLD-1 cells

The expression of GRP78 was knocked down in DLD-1 cells with siRNA. Briefly, the target sequence for the human GRP78 mRNA was 5′-AAGGTTACCCATGCAGTTGTT-3′. The scrambled siRNA sequence was 5′-AAGGTGGTTGTTTTGTTCACT-3′. The GRP78 siRNA and scrambled siRNA were inserted into the pSUPERIOR vector and transfected into the DLD-1 cells. Cells that were successfully transfected and were selected by antibiotic resistance as previously described [17]–[19]. In the present study, the DLD-1 cells transfected with GRP78 siRNA were named GRP78 knockdown cells, and the DLD-1 cells transfected with scrambled siRNA were named scrambled control cells. The expression of GRP78 in the scrambled control cells and the GRP78 knockdown cells was evaluated by western blotting.

### Cell viability assay

Cell viability was evaluated by the trypan blue exclusion assay. Cells (1×10^6^) were cultured in 60-mm tissue culture dishes for 24 h. The culture medium was replaced with new medium, and the cells were exposed to epirubicin for 48 h. After treatment, a mixture was made with 1 part 0.4% trypan blue and 1 part cell suspension. The mixture was then incubated for approximately 3 min at room temperature, a drop of the trypan blue/cell mixture was applied to a hemacytometer and the unstained (viable) and stained (nonviable) cells were separately counted in the hemacytometer.

### DNA damage and cell cycle analysis

DNA damage and the cell cycle were evaluated by PI staining and flow cytometry. Cells (1×10^6^) were cultured in 60-mm tissue culture dishes overnight. The culture medium was replaced with fresh medium, and the cells were treated with epirubicin for 48 h. After treatment, the cells were collected, washed with phosphate-buffered saline (PBS), fixed in PBS-methanol (1∶2, volume/volume) solution, and maintained at 4°C for at least 18 h. After one PBS wash , the cell pellets were stained with a PI solution (PBS, 40 µg/ml PI, and 40 µg/ml DNase-free RNase A) for 30 min at room temperature in the dark and analyzed by a Becton-Dickinson FACScan flow cytometer (Franklin Lakes, NJ). At least 10,000 cells were counted per sample, and the DNA histograms were further evaluated using Modfit software on a PC workstation to calculate the percentage of cells in the various phases of the cell cycle and to quantify the cells with DNA damage (subG_1_ phase).

### Intracellular ROS measurement

The production of intracellular ROS was detected by flow cytometry using DCFH-DA. After treatment, the cells were washed once with PBS, treated with 20 µM DCFH-DA for 30 min in the dark, washed again with PBS, collected by centrifugation, and suspended in PBS. Intracellular ROS levels, which were indicated by the fluorescence of dichlorofluorescein (DCF), were measured through a 530/22-nm barrier filter using a Becton-Dickinson FACScan flow cytometer.

### Nrf2 siRNA transfection

To silence the gene expression of Nrf2, three sets of siRNA oligonucleotides were designed: (1) 5′-GCAUGCUACGUGAUGAAGAtt-3′ (sense) and 5′-UCUUCAUCACGUAGCAUGCtt-3′ (antisense); and (2) 5′-CUCCUACUGUGAUGUGAAAtt-3′ (sense) and 5′-UUUCACAUCACAGUAGGAGtt-3′ (antisense); and (3) 5′-GUGUCAGUAUGUUGAAUCAtt-3′ (sense) and 5′-UGAUUCAACAUACUGACACtt-3′ (antisense). The cells (4×10^5^) were cultured in 60-mm dishes in 5 ml of RPMI-1640 medium complemented by 10% FBS and transfected at 40% confluency by adding Arrest-In transfection agent (Huntsville, AL) and Nrf2 siRNA. Control cells were treated with Arrest-In transfection agent and the scrambled siRNA [5′-UUCUCCGAACGUGUCACGUTT-3′ (sense) and 5′-ACGUGACACGUUCGGAGAATT-3′ (antisense)], which did not lead to the specific degradation of any cellular messages. Cells were rinsed with medium after 25 min of incubation and then maintained in culture for an additional 24 h. The nuclear Nrf2 expression was evaluated by western blotting.

### Akt kinase activity assay

Akt kinase activity was detected using the nonradioactive Akt kinase assay kit. Briefly, cells cultured in 60-mm dishes were treated with 500 ng/ml epirubicin or vehicle as control. After epirubicin treatment, the cells were washed twice with ice-cold PBS and harvested with a cell lysis buffer [20 mM Tris (pH 7.5), 150 mM NaCl, 1 mM EDTA, 1 mM EGTA, 1% Triton, 2.5 mM sodium pyrophosphate, 1 mM β-glycerophosphate, 1 mM Na_3_VO_4_, and 1 µg/ml leupeptin]. The protein content was measured using a Bio-Rad protein assay reagent. Akt kinase was immunoprecipitated from the cell extract (300 µg of protein) using a bead-conjugated phospho-Akt (Ser473) rabbit monoclonal antibody and incubated with gentle rocking overnight at 4°C. The pellets were washed two times with a cell lysis buffer and then washed twice with 500 µ1 of kinase buffer. The pellets were suspended in 50 µl of kinase buffer supplemented with 1 µl of 10 mM ATP and an appropriate quantity of kinase substrate (GSK-3 fusion protein) for 30 min at 30°C. The reaction was terminated with 25 µl of 3× SDS sample buffer. Phosphorylation of GSK-3 fusion protein was detected by western blotting with antiphospho-GSK-3α/β (Ser21/9) rabbit monoclonal antibody.

### Protein phosphatase 2A (PP2A) activity assay

PP2A activity was assayed using the Ser/Thr phosphatase assay kit. Briefly, the cells were lysed with the lysis buffer as suggested by the protocol included in the kit. An aliquot corresponding to 50 µg of protein was used to evaluate the PP2A activity using the protocol from the manufacturer. The absorbance of the reaction solution was measured in 96-well plates at 405 nm in a microplate reader (Bio-Rad, Richmond, CA).

### Western blotting analysis

After treatment, the cells were washed with PBS, resuspended in a protein extraction buffer for 10 min, and centrifuged at 12000× g for 10 min at 4°C to obtain the extracted proteins (supernatant). Protein concentrations were measured with a Bio-Rad protein assay reagent. The extracted cellular proteins were boiled in loading buffer, and an aliquot corresponding to 50–100 µg of protein was separated on a 12% SDS-polyacrylamide gel. After electrophoresis, the proteins were electrotransferred onto a polyvinylidene fluoride transfer membrane. After blotting, the membranes were incubated with various primary antibodies overnight and then washed with PBST solution (0.05% Tween 20 in PBS). Following washing, the secondary antibody, which was labeled with horseradish peroxidase, was added to the membrane for 1 h and then washed with PBST solution (0.05% Tween 20 in PBS). The antigen-antibody complexes were detected by enhanced chemiluminescence (Amersham Pharmacia Biotech, Piscataway, NJ) with a chemiluminescence analyzer.

### Statistical analysis

Data are presented as the mean ± standard deviation from at least three independent experiments and were analyzed using Student's *t* tests. A *P* value of less than 0.05 was considered statistically significant [20].

## Results

### GRP78 knockdown enhanced the cell death and apoptosis induced by epirubicin treatment

**Figure 1:**
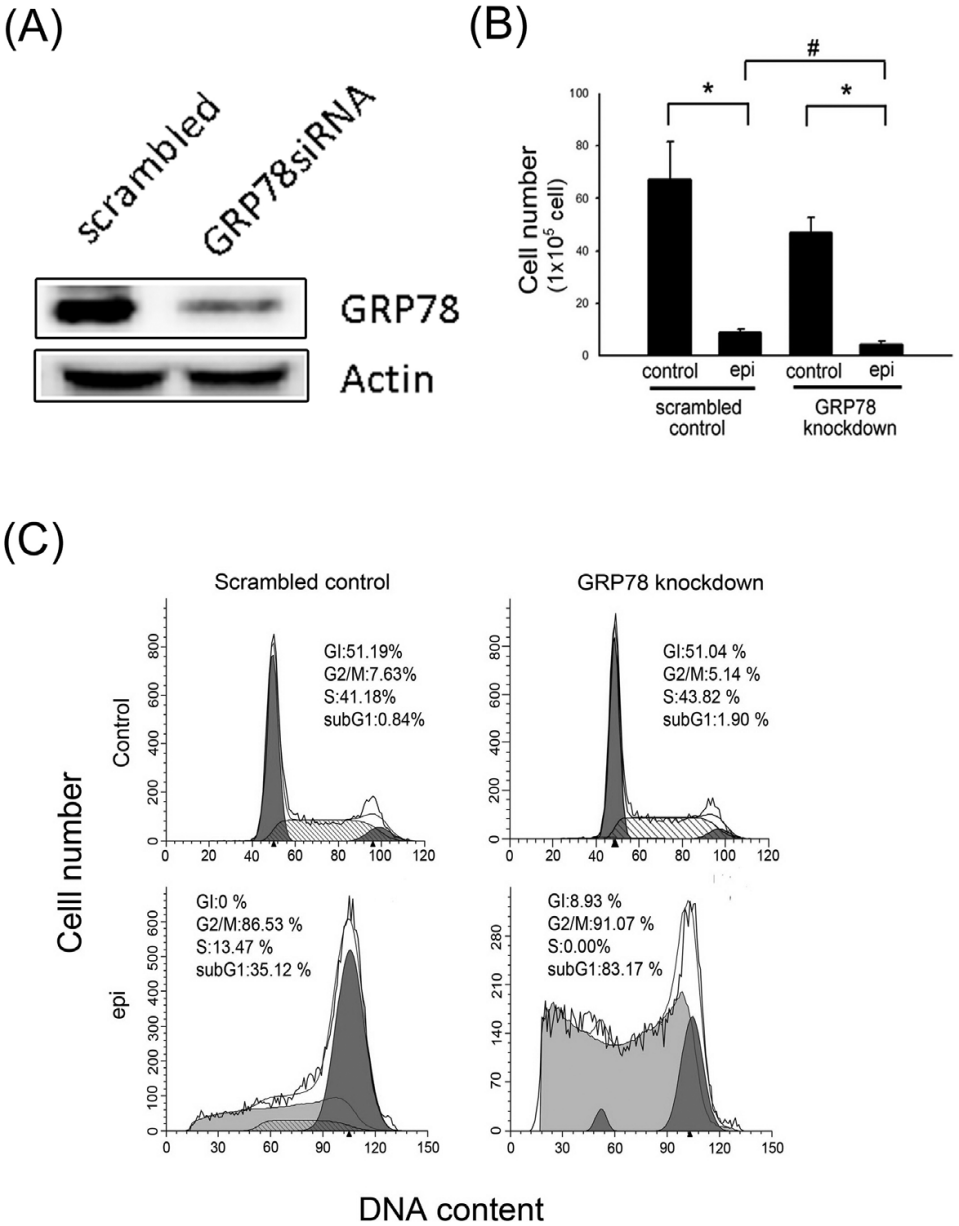
Analysis of GRP78 expression, cell viability and apoptosis. (A) Analysis of GRP78 expression in the scrambled and GRP78 knockdown DLD-1 cells by western blotting. Analysis of (B) cell viability and (C) apoptosis after treatment with vehicle (control) and epirubicin (epi). Scrambled control and GRP78 knockdown DLD-1 cells were treated with epirubicin (500 ng/ml) for 48 h. Cell viability was evaluated by the trypan blue exclusion assay. The percentages of cells that were in the subG_1_ phase (indicated by DNA damage) were determined as outlined in the *Materials and Methods*. The values are represented as the mean ± standard deviation (n = 5–8) of the individual experiments. Significant differences for the control group and the scrambled control group are *P*<0.05 (*) and *P*<0.05 (#), respectively. These experiments were performed at least 3 times, and a representative experiment is presented.

Figure 1A shows the expression of GRP78 in the scrambled control DLD-1 cells and GRP78 knockdown DLD-1 cells. GRP78 knockdown DLD-1 cells showed a lower expression of GRP78 than the scrambled control DLD-1 cells. We further evaluated cell viability and apoptosis after epirubicin treatment in the GRP78 knockdown and scrambled control DLD-1 cells. Figure 1B shows that cell viability was significantly decreased in the GRP78 knockdown cells compared with the scrambled control cells after 48 h of 500 ng/ml epirubicin treatment. In addition, the apoptosis percentage of the GRP78 knockdown cells (83.17%) was much higher than the percentage observed in the scrambled control cells (35.12%) 48 h after epirubicin treatment (Figure 1C). These results suggest that GRP78 is a target protein for epirubicin-induced cell death and apoptosis in DLD-1 colon cancer.

### GRP78 knockdown reduced epirubicin-induced intracellular ROS

**Figure 2:**
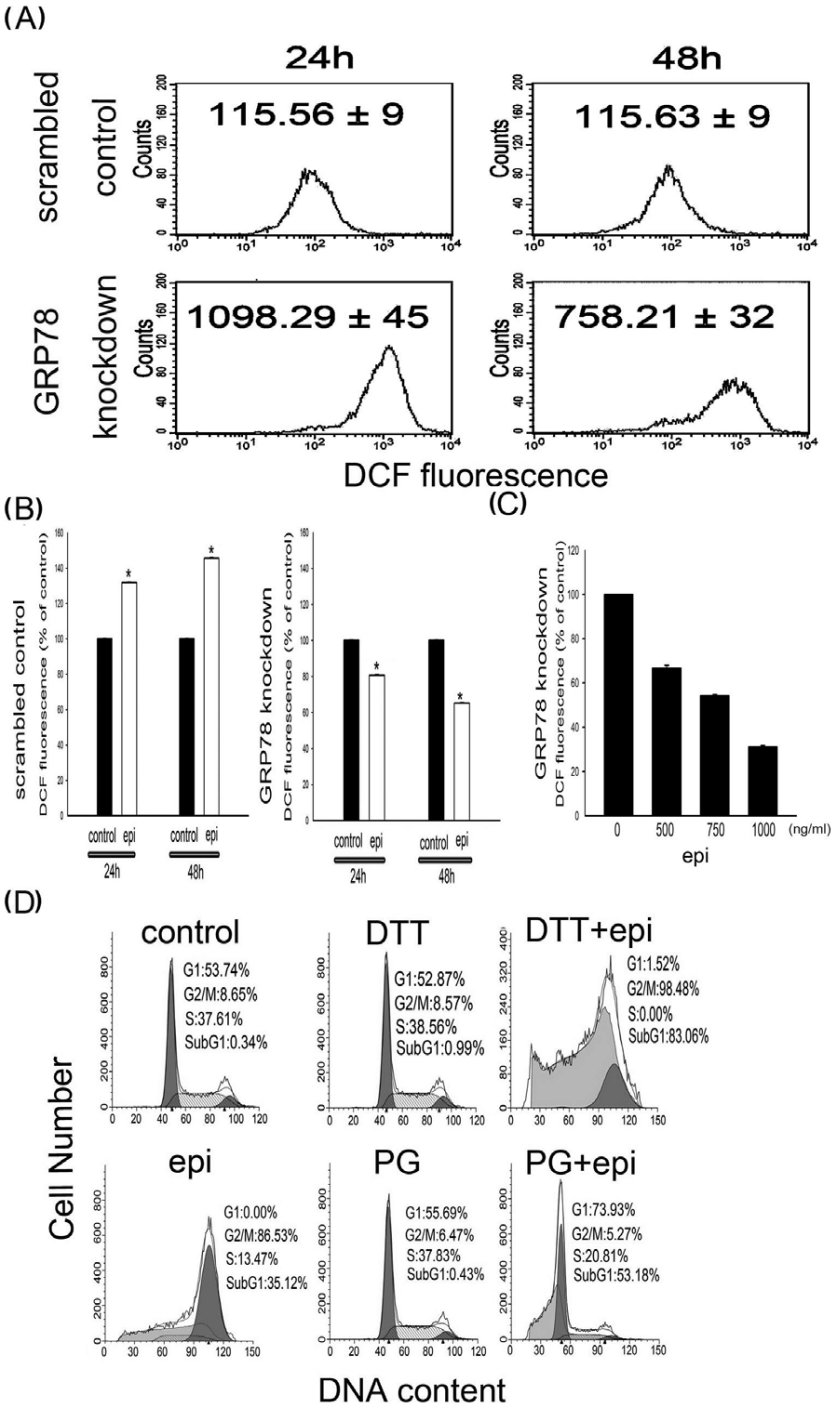
The effect of intracellular oxidative stress on epirubicin (epi) treatment. Scrambled control and GRP78 knockdown DLD-1 cells were (A) cultured for 24 and 48 h or (B) treated with epirubicin (500 ng/ml) for 24 or 48 h. (C) GRP78 knockdown DLD-1 cells were treated with epirubicin (500, 750, 1000 ng/ml) for 48 h. Intracellular ROS were evaluated by DCFH-DA staining and flow cytometry as outlined in the *Materials and Methods*. (D) Scrambled control DLD-1 cells were treated with 500 ng/ml epirubicin (epi) alone, 25 µM propyl gallate (PG) alone, 1 mM dithiothreitol (DTT) alone or epirubicin combined with PG or DTT for 48 h . The percentages of cells that were in the subG_1_ phase (indicated by DNA damage) were determined as outlined in the *Materials and Methods*. The values are represented as the mean ± standard deviation (n = 5–8) of the individual experiments. A significant difference from the control group was set at *P*<0.05 (*).

To evaluate the intracellular oxidative stress, intracellular ROS were evaluated by DCFH-DA staining in the scrambled control cells and the GRP78 knockdown cells. Figure 2A shows that the GRP78 knockdown cells exhibited 7- to 10 -fold greater DCF fluorescence than the scrambled control cells after 24–48 h of culture, which indicated that significant oxidative stress existed in the GRP78 knockdown cells. Treatment of the scrambled control cells with epirubicin resulted in ROS increases of 1.3- and 1.5-fold at 24 and 48 h compared with scrambled control cells without epirubicin (Figure 2B). In contrast, intracellular ROS were decreased by 0.65- and 0.81-fold in the epirubicin-treated GRP78 knockdown cells at 24 and 48 h compared with GRP78 knockdown cells without epirubicin (Figure 2B). The dose of epirubicin over than 500 ng/ml did not increase the ROS in the GRP78 knockdown DLD-1 cells. Epirubicin inhibited the ROS in a dose-dependent manner in the GRP78 knockdown DLD-1 cells (Figure 2C). These results suggest that intracellular ROS reduction might enhance apoptosis in epirubicin-treated scrambled control cells. When the antioxidants PG and DTT, which are used to decrease intracellular ROS, were added to the epirubicin-treated scrambled control cells, apoptosis was increased from 35.12% to 53.18% and 83.06%, respectively (Figure 2D). Interestingly, neither PG nor DTT alone could induce apoptosis in the scrambled control cells.

### Nrf2 is involved in the defense mechanism in the GRP78 knockdown cells

**Figure 3:**
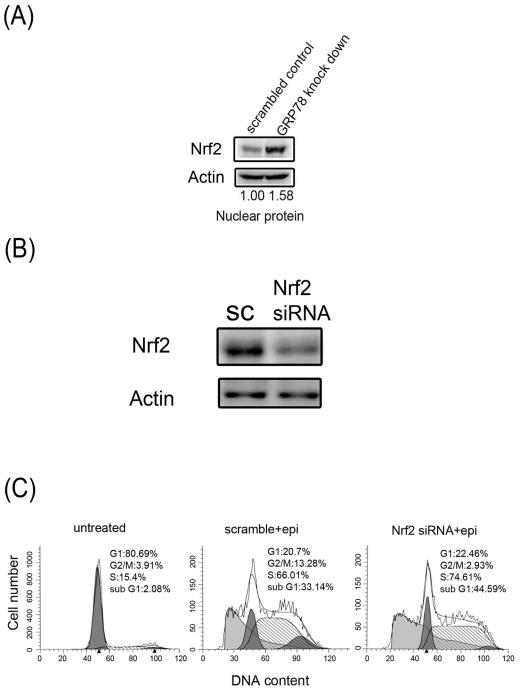
The effect of Nrf2 on epirubicin (epi) treatment. (A) Scrambled control and GRP78 knockdown DLD-1 cells were cultured for 48 h, and nuclear Nrf2 expression was evaluated by western blotting. (B) The GRP78 knockdown DLD-1 cells were pretreated with scrambled siRNA (sc) or Nrf2 siRNA for 24 h, and nuclear Nrf2 expression was evaluated by western blotting. (C) The GRP78 knockdown DLD-1 cells were pretreated with scrambled siRNA (scramble) or Nrf2 siRNA for 24 h and treated with 500 ng/ml epirubicin (epi) for another 48 h. After treatment, the percentage of cells that were in the subG_1_ phase indicated by DNA damage was determined as outlined in the *Materials and Methods*. These experiments were performed at least 3 times, and a representative experiment is presented.

Nrf2 is a transcription factor that responds to intracellular ROS stimulation and translocates to the nucleus by binding the antioxidant response element and transcribing phase II enzymes. We studied Nrf2 nuclear translocation in the scrambled control cells and the GRP78 knockdown cells. Figure 3A shows that Nrf2 nuclear translocation increased approximately 1.6-fold in the GRP78 knockdown cells compared with the scrambled control cells. We also evaluated whether Nrf2 nuclear translocation played a defensive role against epirubicin-induced apoptosis in the GRP78 knockdown cells. The GRP78 knockdown cells were treated with Nrf2 siRNA for 24 h to inhibit Nrf2 expression prior to incubation with epirubicin for 48 h. After the epirubicin incubation, apoptosis was evaluated by flow cytometry. Figure 3B shows that, Nrf2 expression was markedly inhibited by Nrf2 siRNA in the GRP78 knockdown cells, and Figure 3C shows that apoptosis was significantly increased by Nrf2 siRNA treatment in the epirubicin-treated GRP78 knockdown cells, which indicates that Nrf2 provided an important defense mechanism when GRP78 was knocked down in the DLD-1 cells.

### GRP78 knockdown enhanced the epirubicin-mediated inhibition of the Akt pathway

**Figure 4:**
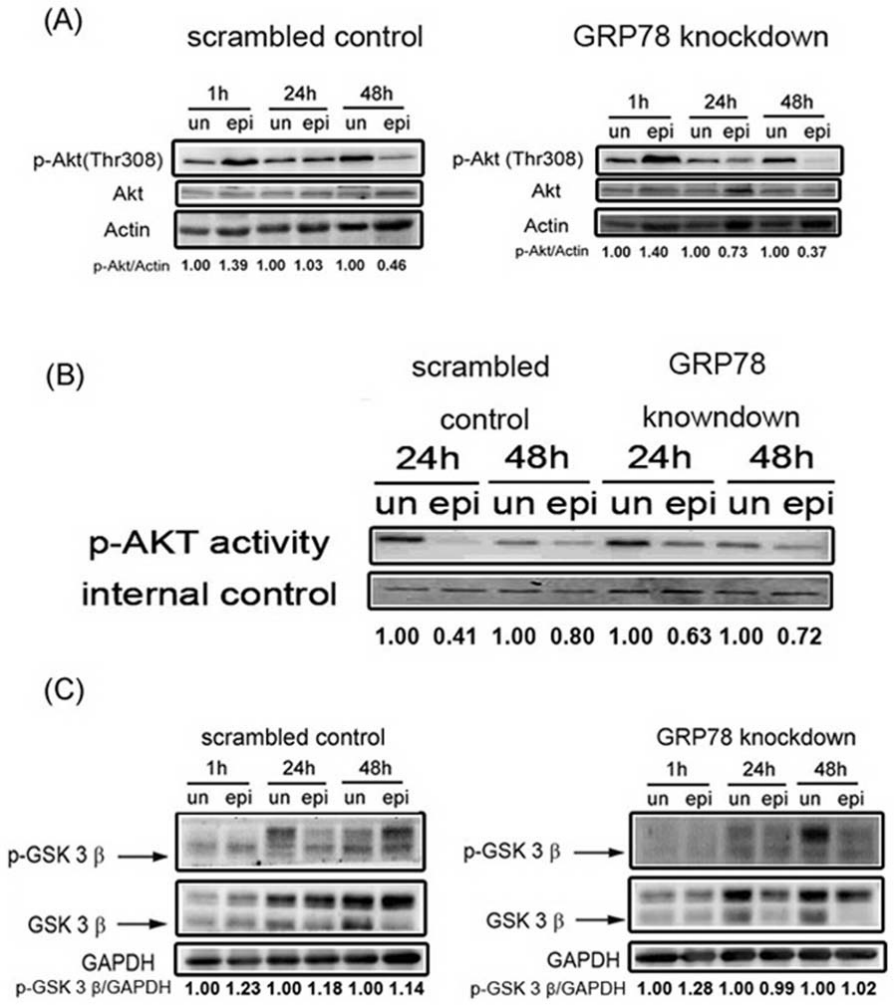
The effect of the Akt pathway on epirubicin (epi) treatment. Scrambled control and GRP78 knockdown DLD-1 cells were treated with epirubicin (500 ng/ml) for the indicated times. The phosphorylation of (A) Akt and (C) GSK 3β was evaluated by western blotting. (B) Akt activity was determined as outlined in the *Materials and Methods*. The protein on the PVDF membrane stained with Coomassie brilliant blue is shown as an internal control. These experiments were performed at least 3 times, and a representative experiment is presented.

There is increasing evidence that the Akt pathway provides a connection between extracellular survival signals and the factors that induce apoptotic events within cells [21]. To investigate whether the Akt pathway is involved in epirubicin-mediated apoptosis, we examined the phosphorylation state of Akt in the scrambled control cells and the GRP78 knockdown cells after epirubicin treatment. Cell lysates were prepared, and the phosphorylation level of Akt was analyzed by western blotting with the anti-phospho-Akt (Thr308) antibody. Akt phosphorylation was decreased to a greater degree in the GRP78 knockdown cells compared with the scrambled control cells after 24 and 48 h of epirubicin treatment (Figure 4A). We also investigated the effects of epirubicin on Akt kinase activity using immunoprecipitation and western blotting techniques. Epirubicin inhibited the phosphorylation level of GSK-3α/3β, (as was seen with the Akt kinase activity) in both the scrambled control cells and the GRP78 knockdown cells, and the decrease in the Akt kinase activity appeared to be enhanced in the GRP78 knockdown cells at 48 h (Figure 4B). Because recent findings have suggested that the GSK-3β protein, which is a key player in stress-responsive apoptosis, is one of the downstream protein substrates for Akt kinase [22], the epirubicin-induced suppression of Akt phosphorylation may inhibit Akt-dependent phosphorylation of GSK-3β. To test this possibility, total protein lysates prepared from epirubicin-treated scrambled control cells and GRP78 knockdown cells were subjected to immunoblotting with a phospho-GSK-3β antibody that specifically recognizes GSK-3β phosphorylated on Ser-9. Scrambled control cells and GRP78 knockdown cells exhibited significant decreases in GSK-3β phosphorylation, and the highest level of inhibition occurred in the GRP78 knockdown cells incubated with epirubicin for 48 h (Figure 4C). These results suggest that the inhibition of the Akt signaling cascade was enhanced by the GRP78 knockdown in epirubicin-treated cells.

### GRP78 knockdown enhanced the epirubicin-mediated downregulation of the downstream targets of the Akt pathway

**Figure 5:**
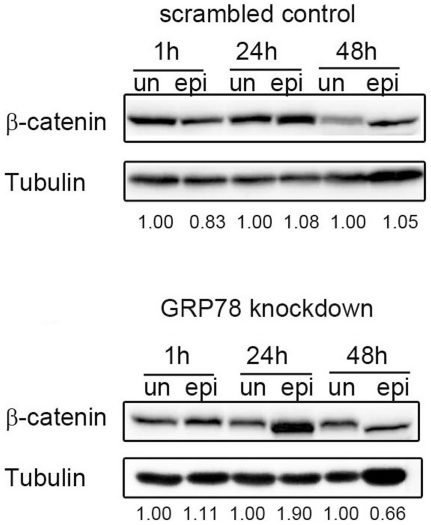
Expression of β-catenin after epirubicin (epi) treatment. Scrambled control and GRP78 knockdown DLD-1 cells were treated with epirubicin (500 ng/ml) for the indicated times, and the expression of β-catenin was evaluated by western blotting. These experiments were performed at least 3 times, and a representative experiment is presented.

We investigated whether inhibition of the Akt signaling pathway had an effect on downstream targets in scrambled control cells and GRP78 knockdown cells treated with epirubicin. β-catenin is a GSK-3β target that is implicated in apoptotic resistance and survival signaling [23], and increased expression of β-catenin has been correlated with increased survival in cancer [23]. An examination of β-catenin expression revealed that it was not significantly changed in the scrambled control cells after 24 or 48 h of epirubicin treatment. A reduction in β-catenin expression, however, was observed after 48 h of epirubicin treatment in the GRP78 knockdown cells (Figure 5).

### GRP78 knockdown enhanced protein phosphatase 2A activity with epirubicin treatment

**Figure 6:**
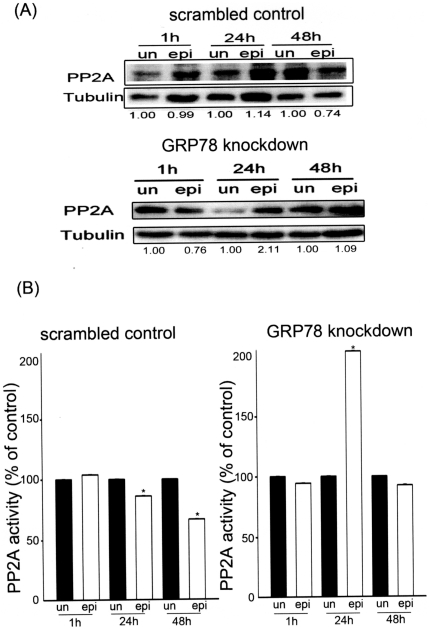
Expression and activity of protein phosphatase 2A (PP2A) after epirubicin (epi) treatment. Scrambled control and GRP78 knockdown DLD-1 cells were treated with epirubicin (500 ng/ml) for the indicated times. (A) The expression of PP2A was evaluated by western blotting, and (B) the activity of PP2A was determined as outlined in the *Materials and Methods*. These experiments were performed at least 3 times, and a representative experiment is presented.

Various studies have demonstrated connections between the Akt pathway and phosphatases. For example, PP2A has been shown to be a key Akt phosphatase that can dephosphorylate Akt at both the Thr 308 and Ser 493 residues and block the Akt pathway [21]. We further examined whether the expression and activity of PP2A was involved in epirubicin-induced apoptosis in the scrambled control cells and the GRP78 knockdown cells. Figure 6A shows that the expression of PP2A decreased in the epirubicin-treated scrambled control cells after 48 h of treatment. Conversely, the expression of PP2A was increased at 24 h and did not decrease at 48 h in the epirubicin-treated GRP78 knockdown cells. The activity of PP2A was slightly decreased at 24 h and significantly decreased at 48 h in the epirubicin-treated scrambled control cells. In contrast, PP2A activity significantly increased approximately 2-fold at 24 h in the epirubicin-treated GRP78 knockdown cells (Figure 6B). PP2A activity did not significantly decrease at 48 h in the epirubicin-treated GRP78 knockdown cells. These experiments demonstrated a clear link between increased PP2A activity and the induction of apoptosis via the inhibition of the Akt pathway in the GRP78 knockdown cells during epirubicin treatment. This highlighted the importance of GRP78 knockdown for the inhibition of DLD-1 cells proliferation.

## Discussion

A common characteristic of many cancers is their augmented levels of ROS. The primary source of these ROS is increased metabolic activity [21]. High ROS levels and Nrf2 nuclear translocation in the GRP78 knockdown cells indicated that GRP78 plays a redox homeostatic role in the DLD-1 cells. In the presence of GRP78, epirubicin only induced a low level of ROS (<2-fold) in DLD-1 cells, and a low level of ROS may result in the activation of specific antioxidant systems in cancer cells that can induce resistance to chemotherapy. Interestingly, the antioxidants DTT and PG both enhanced the apoptotic effect of epirubicin in the scrambled control cells. The reduction of ROS by epirubicin in the GRP78 knockdown cells highlighted the possibility that increased ROS levels in DLD-1 cells may have a direct effect on the cell survival pathways.

GRP78 is a chaperone that exists in the ER. In unstressed cells, the ER transmembrane proteins (PERK and IRE1) are kept in an inactive state via the attachment of GRP78 on their ER-lumenal domains [24]. In general, the lumenal domains of the inactive forms of PERK and IRE1 are comprised of 1∶1 complexes with GRP78 [25]. Once ER stress occurs, GRP78 disjoins and binds with unfolded or misfolded proteins, which enables the homo-oligomerization of PERK, IRE1 or both and results in the autophosphorylation of these enzymes and the activation of their substrates [24]. Nrf2 is a direct substrate of PERK and an effector of PERK-dependent cell survival [26], which explains why the GRP78 knockdown DLD-1 cells expressed a large amount of nuclear Nrf2.

Nrf2 acts as a sensor for oxidative stress and regulates the activation of defensive genes, which leads to the protection of cells against the adverse effects of oxidative stress and promotes cell survival [27]. In many cancer cells, Nrf2 shows pro-tumoral characteristics that are similar to those of identical cytoprotective genes that can increase cancer cell resistance to chemotherapeutic drugs [8]. For example, Fluorouracil stimulates the Nrf2 pathway, which modulates chemosensitivity and induces cytoprotective genes in HT-29 colon cancer cells [9]. Nrf2 is a crucial transcription factor that controls a protective response against toxic insults from an extensive spectrum of chemical substances [25]. In recent years, studies have demonstrated the role of Nrf2 in protecting against both new and current chemotherapeutic drugs in blood cancers [8]. Nrf2 overexpression has also been observed in pancreatic cancer [7]. In addition, a study has shown that the stable overexpression of Nrf2 in cancer cells induces an enhanced level of resistance to chemotherapeutic agents, including doxorubicin, etoposide and cisplatin [25]. Other reports have suggested that the strategy of using Nrf2 inhibitors to augment the efficacy of chemotherapeutic agents is not restricted to anticancer drugs or certain cancer types. Indeed, Nrf2 inhibitors can be utilized during chemotherapy to treat many cancer types [25]. The present study revealed that apoptosis was enhanced by epirubicin treatment in the Nrf2 siRNA-treated GRP78 knockdown cells, which suggests that Nrf2 plays a critical defensive role in the GRP78 knockdown cells.

A recent study revealed that the silencing of GRP78 gene expression by RNAi suppressed the activation of Akt (i.e., Thr 308 phosphorylation) in 1-LN human prostate cancer cells [28]. In addition, another study showed that the phosphorylation of Akt was decreased in astrocytoma after transfection with GRP78 siRNA [29]. Lin et al. (2011) recently reported that Akt was a downstream target of GRP78 in intervening cisplatin resistance in ER-stress-tolerant human lung cancer cells [30]. These findings indicated that the GRP78-directed Akt pathway plays an important role in cancer survival. The present results showed that GRP78 knockdown enhanced the inhibition of Akt activation with epirubicin treatment in DLD-1 cells, which led to the inhibition of downstream Akt substrates. The results of the present study suggest that GRP78 knockdown may be a useful addition to the arsenal of chemtherapeutic drugs that can be applied to promote death in cancer cells by inhibiting the Akt survival signaling pathway.

The Akt pathway is constitutively active in many cancers, such as skin, breast, prostate and colon cancer. After Akt activation, Akt phosphorylates key survival proteins, such as the transcription factor regulator GSK-3β. The phosphorylation of GSK-3β decreases the susceptibility to apoptotic stresses in cancer cells. The Akt signaling pathway is transduced via phosphorylation, and phosphatases are the most important negative regulators. One such phosphatase is PP2A, which can catalyze the opposite reaction to Akt. Recently, a study demonstrated that exogenous hydrogen peroxide could inhibit PP2A, and this resulted in the activation of the Akt pathway [31]. Another study demonstrated that cadmium-induced ROS also inhibited PP2A, which led to activation of Erk1/2, JNK and apoptosis [32]. In line with these observations, our data demonstrated that epirubicin treatment in GRP78 knockdown DLD-1 cells decreased intracellular ROS and activated PP2A. In addition, we showed that GSK-3β phosphorylation and β-catenin expression were inhibited by epirubicin in the GRP78 knockdown cells. Taken together, these results indicate an important pathway involving ROS reduction and PP2A activation, where epirubicin inhibits the Akt survival pathway and enhances the apoptotic effect in the GRP78 knockdown cells.

Many *in vitro* studies have demonstrated that treating cancer cells with compounds that have redox-lowering properties increases their apoptotic susceptibility [21]. This suggests a great potential for the use of antioxidants to decrease anti-apoptotic pathways that are activated by low levels of ROS in cancer cells. This finding is in agreement with our observation that the antioxidants, DTT and PG both enhanced apoptosis in the GRP78 scrambled cells. These redox-operating compounds can be considered for use in combination with current clinical chemotherapeutic drugs to attain beneficial effects.

Epirubicin's mechanism of cytotoxicity appears to involve the production of ROS via significant reduction of catalase, superoxide dismutase and cytosolic glutathione levels [33]. Several oxidase enzymes, including nicotinamide-adenine dinucleotide phosphate (NADPH) oxidase, xanthine oxidase, uncoupled endothelial NO synthase (eNOS), cyclooxygenase, glucose oxidase, and lipooxygenase can produce intracellular ROS. The mitochondrial electron transport also provides the source of intracellular ROS [34]. In GRP78 knockdown DLD-1 cells, epirubicin might damage or inhibit these ROS generated enzymes or induce mitochondrial dysfunction and then reduce intracellular ROS. On the other hand, epirubicin might increase superoxide dismutase, catalase or cytosolic glutathione levels to reduce intracellular ROS in GRP78 knockdown DLD-1 cells.

Our experimental results indicate that epirubicin increased apoptosis in GRP78 knonkdown DLD-1 cells. In clinical application, we can consider to use gene therapy to knockdown the GRP78 expression in colon cancer cells accompany with epirubicin chemotherapy. In addition, (-)-epigallocatechin gallate (EGCG) has been found to directly interact with GRP78 at the ATP-binding site of protein and regulate its function by competing with ATP binding, resulting in the inhibition of ATPase activity. EGCG binding results in the conversion of GRP78 from its active monomer to the inactive dimer and oligomer forms [35]. The combination of epirubicin and EGCG chemotherapy also can be considered in clinical application.

In conclusion, GRP78 knockdown can change the oxidative stress status of cancer cells. In the present study, this involved increased intracellular ROS and Nrf2 nuclear translocation in DLD-1 cells. Epirubicin enhanced apoptosis by reducing intracellular ROS, activating PP2A and inhibiting the Akt survival pathway in the GRP78 knockdown DLD-1 cells.
